# Lipidomic Analysis of Plasma from Healthy Men and Women Shows Phospholipid Class and Molecular Species Differences between Sexes

**DOI:** 10.1002/lipd.12293

**Published:** 2020-12-07

**Authors:** Annette L. West, Louise V. Michaelson, Elizabeth A. Miles, Richard P. Haslam, Karen A. Lillycrop, Ramona Georgescu, Lihua Han, Johnathan A. Napier, Philip C. Calder, Graham C. Burdge

**Affiliations:** ^1^ School of Human Development and Health, Faculty of Medicine, Southampton General Hospital University of Southampton Tremona Road Southampton SO16 6YD UK; ^2^ Department of Plant Sciences Rothamsted Research West Common Harpenden AL5 2JQ UK; ^3^ Centre for Biological Sciences, Faculty of Natural and Environmental Sciences University of Southampton University Road Southampton SO17 1BJ UK; ^4^ NIHR Southampton Biomedical Research Centre University Hospital Southampton NHS Foundation Trust and University of Southampton Tremona Road Southampton SO16 6YD UK

**Keywords:** Arachidonic acid, Docosahexaenoic acid, Phospholipid molecular species, Plasma, Sex differences

## Abstract

The phospholipid composition of lipoproteins is determined by the specificity of hepatic phospholipid biosynthesis. Plasma phospholipid 20:4n‐6 and 22:6n‐3 concentrations are higher in women than in men. We used this sex difference in a lipidomics analysis of the impact of endocrine factors on the phospholipid class and molecular species composition of fasting plasma from young men and women. Diester species predominated in all lipid classes measured. 20/54 Phosphatidylcholine (PtdCho) species were alkyl ester, 15/48 phosphatidylethanolamine (PtdEtn) species were alkyl ester, and 12/48 PtdEtn species were alkenyl ester. There were no significant differences between sexes in the proportions of alkyl PtdCho species. The proportion of alkyl ester PtdEtn species was greater in women than men, while the proportion of alkenyl ester PtdEtn species was greater in men than women. None of the phosphatidylinositol (PtdIns) or phosphatidylserine (PtdSer) molecular species contained ether‐linked fatty acids. The proportion of PtdCho16:0_22:6, and the proportions of PtdEtn O‐16:0_20:4 and PtdEtn O‐18:2_20:4 were greater in women than men. There were no sex differences in PtdIns and PtdSer molecular species compositions. These findings show that plasma phospholipids can be modified by sex. Such differences in lipoprotein phospholipid composition could contribute to sexual dimorphism in patterns of health and disease.

**Abbreviations**
DAG: diacylglycerol
HR/AM: high resolution/accurate mass
LC/MS: liquid chromatography/mass spectrometry
LPAT: lyso phospholipid acyltransferase
MTBE: methyl‐tert‐butyl‐ether
PtdCho: phosphatidylcholine
PtdEtn: phosphatidylethanolamine
PtdIns: phosphatidylinositol
PLA: phospholipase A
PtdSer: phosphatidylserine
PUFA: polyunsaturated fatty acid
UPLC: ultra‐performance liquid chromatography

## Introduction

The molecular species composition of phospholipids differs between tissues and phospholipid classes (Inoue et al., 2017) and is regulated by biochemical and genetic processes (Zhang and Rock, 2008). The combination of fatty acids bound at the *sn*‐1 and *sn*‐2 positions, at least in part, determines the biophysical properties of cell membranes which influence the activities of integral membrane proteins (Ces, 2006; Dymond et al., 2013) and provide substrates for signaling processes (Heung and Postle, 1995a, b). Thus, factors that confer membrane phospholipid homeostasis are important for maintaining cell function (Calder, 2008). Fasting plasma phospholipid molecular species composition primarily reflects that of the liver and hepatic phospholipid synthesis (Burdge et al., 1994; Chalil et al., 2018; Pynn et al., 2011). Therefore, plasma phospholipid composition can be a proxy for hepatic phospholipid composition and can provide insights into phospholipid metabolism in the liver.

Synthesis of phosphatidylcholine (PtdCho), which is the predominant component of the lipoprotein phospholipid monolayer, is required for the formation and secretion of very low density lipoproteins (VLDL) (Yao and Vance, 1988). Inhibition of PtdCho synthesis can alter the phospholipid composition and structure of VLDL particles (Fast and Vance, 1995). Hepatic PtdCho and phosphatidylethanolamine (PtdEtn) are synthesized *via* the Kennedy pathway from CDP‐choline and CDP‐ethanolamine, respectively (Gibellini and Smith, 2010; Kanoh and Ohno, 1975; Weiss et al., 1958). The composition of newly synthesized phospholipids reflects that of the metabolically and compositionally distinct diacylglycerol (DAG) substrate pools (Burdge et al., 1994; Rustow et al., 1985; Rustow and Kunze, 1985). Approximately 30% of hepatic PtdCho synthesis occurs by the PtdEtn N‐methylation pathway (Sundler and Akesson, 1975; Vance et al., 1997), which yields mainly polyunsaturated fatty acid (PUFA)‐containing PtdCho species (Burdge et al., 1994; Chalil et al., 2018). The minor plasma phospholipids, namely phosphatidylinositol (PtdIns), and phosphatidylserine (PtdSer), are synthesized by PtdIns synthase *via* CDP‐DAG (Blunsom and Cockcroft, 2020) and by base exchange with PtdCho (PtdSer synthase I) or with PtdEtn (PtdSer synthase II) (Leventis and Grinstein, 2010), respectively. The roles of PtdIns and PtdSer in lipoprotein function are not known. Newly synthesized phospholipids can undergo acyl remodeling processes *via* the Lands cycle (Lands, 1960) to generate the molecular species composition of the mature phospholipid pool. This involves phospholipase A_2_ (PLA_2_) or PLA_1_, and lyso phospholipid acyltransferase (LPAT) activities (Schmid et al., 1991; Wang and Tontonoz, 2019). Specificity of acyl remodeling, and hence the composition of the final phospholipid pool, is conferred by LPAT substrate selectivity (Wang and Tontonoz, 2019). For example, in rat and guinea pig liver, newly synthesized PtdCho undergoes acyl exchange at the *sn*‐1 position, which results in partial conversion of *sn*‐1 16:0 to *sn*‐2 18:0 species (Burdge et al., 1993, 1994).

It is well established that phospholipid biosynthesis and membrane composition are under genetic and biochemical control (Sugimoto et al., 2008). However, the molecular species composition of hepatic and plasma phospholipids appears to also be under endocrine control. In pregnant women, increasing gestational age is accompanied by changes in plasma PtdCho 18:1n‐9, 18:2n‐6, 20:4n‐6 and 22:6n‐3 concentrations (Al et al., 2000; Meyer et al., 2016), specifically in *sn*‐1 16:0 or *sn*‐1 18:1n‐9 PtdCho molecular species (Postle et al., 1995). Similarly, pregnancy in rats involves a differential increase in hepatic and plasma PtdCho16:0/22:6 and 16:0/20:4 concentrations compared to 18:0/22:6 and 18:0/20:4 concentrations (Burdge et al., 1994; Chalil et al., 2018; Childs et al., 2012), which reflects the increased *sn*‐1 16:0 content of the DAG substrate pools destined for PtdCho and PtdEtn synthesis, and reduced flux through the acyl remodeling pathway. Together adaptations result in enrichment *sn*‐1 16:0 PtdCho and PtdEtn molecular species without a change in the specificity of the Lands cycle (Burdge et al., 1994). Administration of sex hormones to gonadectomized rats induced reciprocal changes in the PUFA content of liver phospholipids (Eden et al., 1987). One implication of these findings is that regulation of hepatic and plasma phospholipid compositions may be important for sex‐related tissue and lipoprotein functions.

The proportions of 20:4n‐6 and 22:6n‐3 are typically 20% higher in total fasting plasma lipids and in phospholipids from women compared to men (Lohner et al., 2013). This sex difference is independent of dietary fatty acid intakes (Bakewell et al., 2006), and may be explained, at least in part, by greater capacity for PUFA biosynthesis in young women than in men (Burdge et al., 2002; Burdge and Wootton, 2002). Here, we used the differences in the proportions and/or concentrations of PUFA between men and women (Lohner et al., 2013) as a model to investigate the specificity of sex‐related differences in plasma phospholipid composition. We analyzed the PtdCho, PtdEtn, PtdIns, and PtdSer molecular species compositions of plasma collected from fasting healthy men and women.

## Materials and Methods

### Ethics Statement

The study was reviewed by the South Central—Hampshire B Research Ethics Committee (REC reference 15/SC/0627) who approved the study and participants gave written informed consent. The study is registered at ClinicalTrials.gov (Identifier: NCT03477045).

### Participants and Sample Collection

Blood samples were collected at baseline from a postprandial lipid metabolism study that has been reported in detail previously (West et al., 2019). Briefly, participants were 10 healthy men aged 25 ± 1 year and 10 healthy women aged 25 ± 1 year, both with body‐mass‐index of 24 ± 1 kg/m^2^. Participants had blood pressure, and fasting total triacylglycerol, cholesterol, and glucose concentrations within normal ranges (West et al., 2019). The habitual diets of the participants were not matched or modified for the purpose of the study. Volunteers were excluded if they consumed oily fish more than once per week, took fish oil or other dietary supplements and/or smoked tobacco. Venous blood was collected at approximately 8:00 AM after fasting overnight for approximately 12 h into tubes containing lithium heparin anticoagulant. Cells were removed from blood by centrifugation at 4°C (West et al., 2016) and the plasma then stored at −80°C.

### Analysis of Plasma Phospholipid Molecular Species Compositions

The methods for lipid extraction and analysis by liquid chromatography/mass spectrometry have been described previously (West et al., 2020). Briefly, total lipids were extracted from plasma (200 μL) with methyl‐tert‐butyl‐ether (MTBE) (Matyash et al., 2008). The organic phase was dried under a stream of nitrogen, dissolved in chloroform/methanol (9:1, v/v) and stored at −20°C in a nitrogen atmosphere.

Phospholipid molecular species compositions were analyzed by high resolution/accurate mass (HR/AM) lipidomics using a Vanquish—Q Exactive Plus UPLC‐MS/MS system (Thermo Fisher Scientific, Newport, Gwent, UK) as described (West et al., 2020). Briefly, plasma total lipids were resuspended in 500 μL chloroform: methanol (1:1, v/v) and along with the internal standard PtdCho24:1/24:1 (0.857 μM). The autosampler tray was maintained at 10 °C and 20 μL of each sample was injected into the ultra‐performance liquid chromatography (UPLC)/MS system. Chromatographic separation was achieved using an Accucore C18 (2.1 μm × 150 mm, 2.6 mm) column (Thermo Fisher Scientific) at 35°C with a flow rate of 400 μL/min. An elution gradient was applied to column of mobile phase A (10 mM ammonium formate in 50% acetonitrile +0.1% (v/v) formic acid) and B (2 mM ammonium formate in acetonitrile: propan‐2‐ol: water (10:88:2 v/v/v) + 0.02% (v/v) formic acid) over 28 min; starting at 35% (v/v) B, then 60% B at 4 min, 85% B at 12 min, and 100% (v/v) B at 21 min which was held for 3 min before equilibrating for 4 min at 35% B prior to the next run. The elution system was based on Bird et al. (2011), but was modified such that ammonium formate concentration was varied in order to optimize the ionization conditions for the different lipid classes (Constantinou et al., 2020).

The Thermo Q Exactive HESI II sweep plate set in position C. Conditions were adjusted for separate positive and negative runs; replicate runs of samples in a single polarity increased the number of identifications. LC/MS at 140K full scan data HCD MS2 experiments (35K resolution) were performed in positive and negative ion modes. Full Scan was operated at 140,000 resolution across *m*/*z* 150–1200, with Top 15 selection MS/MS at 35,000 resolution. The stepped collision energy used was 25, 30, 40, and the dynamic exclusion set to 8s. The sheath gas set to 60 (arbitrary units), auxillary gas 20, sweep gas 1, spray voltage 3 KV in positive ion mode and 3.2KV negative ion mode, S‐lens at 50 for +ve ion and 60 in ‐ve ion, capillary temperature 320 °C and aux gas heater set to 370°C. Automatic gain targets of 1^6^ (full scan MS) and 1^5^ (MS/MS) were used. This protocol was optimized to fragment as many of the detected peaks as possible to allow them to be identified by comparison to MS/MS libraries. The samples were run on the instrument in a random order and analyzed blind using a number coding system. Blanks were run every four samples to check for carry over and background effects. The first sample of the batch was rerun at the end of the batch to check for any difference in performance.

LipidSearch 4.2 experimental workflow (Thermo Fisher Scientific) was used for lipid characterization and putative lipid species were identified separately from positive or negative ion adducts. The data for each replicate were aligned within a chromatographic retention time window by combining the positive and negative ion annotations and merging these into a single annotation. The MS/MS fragmentation spectrum was used to identify the class and fatty chain composition of the lipid species. Experimental MS/MS spectra were searched against all lipid classes in the LipidSearch database using a precursor mass tolerance of 5 ppm and a product mass tolerance of 8 ppm, and the quality of the annotation was graded A‐D by the software. Only those graded A or B were used in this analysis. In grade A, all fatty acyl chains and the class were identified; in grade B, some fatty acyl chains and the class were identified. Final assignment was based on UPLC separation. Any isomers that could not be adequately separated based on retention time were then subject to additional fragmentation analysis of the sodium adducts in positive ionization mode and of the deprotonated species in negative ionization mode (Huynh et al., 2019).

Peak areas were normalized to the internal standard; peak areas corresponding to individual phospholipid molecular species were expressed as a proportion of the total species in each lipid class. Raw lipid molecular species composition data are deposited at EMBL‐EBI MetaboLights database (http://www.ebi.ac.uk/metabolights; Identifier: MTBLS1348) (Haug et al., 2013).

Putative assignment of some fatty acids to *sn*‐1 and *sn*‐2 positions was based on previous analyses of human plasma phospholipid molecular species (Heung and Postle, 1995b; Postle et al., 1995; Pynn et al., 2011; West et al., 2020). However, since the positions of fatty acids were not determined for all the phospholipid molecular species reported here, combinations of fatty acids are separated by an underscore (Murphy, 2018) and do not indicate *sn* positions. Lysophospholipids and phosphatidic acid were excluded from the analyses because they arise as a result of lipase actions in plasma or as a consequence of sample degradation, rather than being the product of hepatic synthesis. Phosphatidic acid was not detected in these samples.

The samples were analyzed blind using a number code system and in a random order. Blanks were run every four samples to check for carry over and background effects. The first sample of the batch was rerun at the end of the batch to check for any difference in the technical performance of the instrument and sample stability in the autosampler. The internal standard, PtdCho 24:1/24:1, was used to correct for instrument analytical variation.

### Statistical Analysis

Molecular species that individually contributed less than 0.01% of total molecular species in each class were excluded from analysis for differences between sexes. Analysis of residuals by the Shapiro‐Wilks test showed that all data approximated a normal distribution and hence are expressed as mean ± standard error of the mean (SEM). Comparisons between men and women were by Student's unpaired *t*‐test with adjustment for multiple testing using the Holm‐Šídák method. Statistical significance after adjustment was assumed at *p* < 0.05.

## Results

### Effect of Sex on the Proportions of PtdCho Molecular Species in Plasma

**Fig 1:**
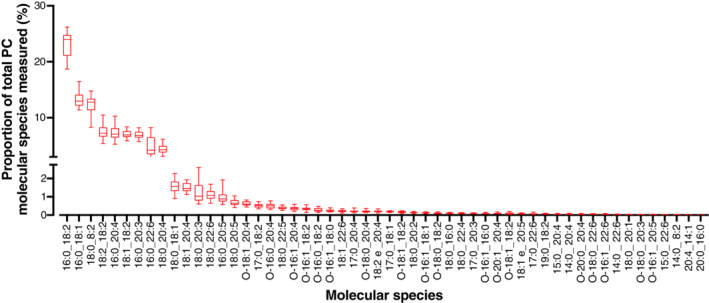
Plasma phosphatidylcholine molecular species composition. Values are proportions in descending order of individual molecular species for n = 20 participants (men plus women, n = 10 per sex). Data points represent individual participants. Bar = mean (range). O,*sn*‐1 alkyl,*sn*‐2 ester species; P,*sn*‐1 alkenyl,*sn*‐2 ester species

Thirty‐four diester PtdCho molecular species and 20 alkyl‐ester PtdCho molecular species were identified consistently in plasma from men and women (Tables 1 and 2). The molecular species profile of plasma PtdCho for all participants is shown in Fig. 1. *sn*‐1,2 ester‐linked PtdCho species accounted for greater than 95% of all PtdCho molecular species in men and women (Table 1). The most abundant species, namely, PtdCho16:0_18:2, PtdCho16:0_18:1, and PtdCho18:0_18:2 together accounted for 49.3 ± 1.4% and 47.7 ± 1.2% of total PtdCho molecular species in men and women, respectively (Table 1). Eighteen diester PtdCho species in women and 17 diester PtdCho species in men each contributed less than 0.5% each of the total PtdCho species, and combined accounted for less than 2% of total PtdCho species in both sexes (Table 1). Comparisons between men and women showed that the proportion of PtdCho16:0_22:6 was significantly greater (64.4%) in women than in men (Table 1). Linear regression analysis showed that differences in sex accounted for 41.9% (adjusted *r* ^2^ = 0.419; *p* = 0.001) of the variation in the proportion of PtdCho16:0_22:6. The proportions of other PtdCho molecular species did not differ significantly between men and women.

**Table 1:** Plasma phosphatidylcholine *sn*‐1,2 ester linked molecular species composition in men and women

	Proportion of total PtdCho molecular species (%)		*t*‐Test	
Molecular species	Men	Women	*p*‐value	Adjusted *p*‐value
PtdCho14:0_18:2	0.01 ± 0.01	0.01 ± 0.01	0.184	>0.999
PtdCho14:0_20:4	0.04 ± 0.01	0.04 ± 0.01	0.700	>0.999
PtdCho14:0_22:6	0.03 ± 0.01	0.03 ± 0.01	0.443	>0.999
PtdCho15:0_20:4	0.04 ± 0.01	0.04 ± 0.01	0.804	0.131
PtdCho15:0_22:6	0.01 ± 0.01	0.02 ± 0.01	0.196	>0.999
PtdCho16:0_18:1	12.89 ± 0.33	13.41 ± 0.53	0.413	>0.999
PtdCho16:0_18:2	23.80 ± 0.81	22.30 ± 0.60	0.155	>0.999
PtdCho16:0_20:3	7.05 ± 0.23	6.73 ± 0.19	0.305	>0.999
PtdCho16:0_20:4	7.29 ± 0.43	7.50 ± 0.41	0.728	>0.999
PtdCho16:0_20:5	0.96 ± 0.12	0.91 ± 0.06	0.706	>0.999
PtdCho16:0_22:6	3.71 ± 0.33	6.10 ± 0.53	0.002	0.013
PtdCho17:0_18:1	0.21 ± 0.01	0.17 ± 0.01	0.004	>0.999
PtdCho17:0_18:2	0.60 ± 0.03	0.43 ± 0.01	0.001	>0.999
PtdCho17:0_20:3	0.10 ± 0.0	0.09 ± 0.01	0.109	>0.230
PtdCho17:0_20:4	0.22 ± 0.02	0.20 ± 0.02	0.558	>0.999
PtdCho17:0_22:6	0.08 ± 0.01	0.05 ± 0.01	0.071	0.963
PtdCho18:0_16:0	0.11 ± 0.01	0.09 ± 0.01	0.153	>0.999
PtdCho18:0_18:1	1.54 ± 0.12	1.55 ± 0.12	0.990	>0.999
PtdCho18:0_18:2	12.65 ± 0.56	11.75 ± 0.56	0.275	>0.999
PtdCho18:0_20:1	0.02 ± 0.01	0.02 ± 0.01	0.236	>0.999
PtdCho18:0_20:2	0.14 ± 0.01	0.13 ± 0.01	0.383	0.969
PtdCho18:0_20:3	1.14 ± 0.13	1.29 ± 0.19	0.511	0.927
PtdCho18:0_20:4	4.19 ± 0.28	4.51 ± 0.22	0.374	0.832
PtdCho18:0_20:5	0.66 ± 0.06	0.70 ± 0.04	0.634	0.996
PtdCho18:0_22:4	0.09 ± 0.01	0.10 ± 0.01	0.435	>0.999
PtdCho18:0_22:5	0.38 ± 0.03	0.39 ± 0.03	0.100	0.826
PtdCho18:0_22:6	1.07 ± 0.09	1.12 ± 0.09	0.728	>0.999
PtdCho18:1_18:2	7.19 ± 0.23	6.84 ± 0.19	0.254	>0.999
PtdCho18:1_20:4	1.58 ± 0.08	1.43 ± 0.06	0.179	>0.999
PtdCho18:1_22:6	0.25 ± 0.02	0.17 ± 0.01	0.007	>0.999
PtdCho18:2_18:2	7.47 ± 0.44	7.65 ± 0.42	0.759	>0.999
PtdCho19:0_18:2	0.06 ± 0.01	0.05 ± 0.01	0.012	>0.999
PtdCho20:0_16:0	0.01 ± 0.01	0.01 ± 0.01	0.818	>0.999
PtdCho20:4_14:1	0.01 ± 0.01	0.01 ± 0.01	0.908	>0.999
Total	95.60 ± 0.22	95.86 ± 0.21	0.401	>0.999

Values are mean ± SEM (n = 10 per sex) proportions of individual molecular species in total plasma PtdCho. Comparisons of means between sexes were by Student's unpaired *t*‐test after adjustment for multiple testing using the Holm‐Šídák method. Molecular species are ranked by increasing number of carbons and degree of unsaturation of the putative *sn*‐1 fatty acid.

**Table 2:** Plasma phosphatidylcholine *sn*‐1 alkyl, *sn*‐2 ester‐linked molecular species composition in men and women

	Proportion of total PtdCho molecular species (%)		*t*‐Test
Molecular species	Men	Women	*p*‐value
PtdCho O‐16:0_18:2	0.27 ± 0.03	0.25 ± 0.03	0.719
PtdCho O‐16:0_20:4	0.48 ± 0.05	0.52 ± 0.04	0.432
PtdCho O‐16:1_16:0	0.09 ± 0.01	0.09 ± 0.01	0.829
PtdCho O‐16:1_18:0	0.25 ± 0.02	0.22 ± 0.02	0.296
PtdCho O‐16:1_18:1	0.13 ± 0.01	0.13 ± 0.02	0.990
PtdCho O‐16:1_20:5	0.02 ± 0.00	0.02 ± 0.01	0.871
PtdCho O‐16:1_22:6	0.04 ± 0.01	0.04 ± 0.01	0.839
PtdCho O‐16:1_18:2	0.37 ± 0.03	0.30 ± 0.02	0.071
PtdCho O‐16:1_20:4	0.37 ± 0.04	0.34 ± 0.03	0.563
PtdCho O‐18:0_18:2	0.11 ± 0.01	0.09 ± 0.01	0.248
PtdCho O‐18:0_20:3	0.02 ± 0.00	0.02 ± 0.00	0.681
PtdCho O‐18:0_20:4	0.20 ± 0.02	0.21 ± 0.02	0.828
PtdCho O‐18:0_22:6	0.04 ± 0.00	0.04 ± 0.01	0.921
PtdCho O‐18:1_18:2	0.17 ± 0.01	0.15 ± 0.02	0.421
PtdCho O‐18:1_18:2	0.08 ± 0.02	0.07 ± 0.02	0.888
PtdCho O‐18:1_20:4	0.64 ± 0.04	0.59 ± 0.04	0.389
PtdCho O‐18:1_20:5	0.07 ± 0.01	0.07 ± 0.01	0.969
PtdCho O‐18:2_20:4	0.22 ± 0.02	0.19 ± 0.01	0.196
PtdCho O‐20:0_20:4	0.04 ± 0.01	0.04 ± 0.00	0.967
PtdCho O‐20:1_20:4	0.10 ± 0.01	0.09 ± 0.01	0.424
Total	3.72 ± 0.26	3.48 ± 0.23	0.500

Values are mean ± SEM (n = 10 per sex) proportions of individual molecular species in total plasma PtdCho. Comparisons of means between sexes were by Student's unpaired *t*‐test with adjustment for multiple testing using the Holm‐Šídák method (all adjusted *p*‐values were nonsignificant and are not shown). Molecular species are ranked by increasing number of carbons and degree of unsaturation of the putative *sn*‐1 fatty acid.

Alkyl‐ester PtdCho species each accounted for less than 4% of total PtdCho molecular species (Table 2). PtdCho O‐18:1_20:4, PtdCho O‐16:0_20:4, and PtdCho O‐16:1_20:4 were the most abundant alkyl‐ester PtdCho species in men and women, and accounted for less than 1.5% of total PtdCho molecular species in both sexes. There were no significant differences in the proportions of alkyl‐ester PtdCho species between men and women (Table 2).

### Effect of Sex on the Proportions of PtdEtn Molecular Species in Plasma

**Fig 2:**
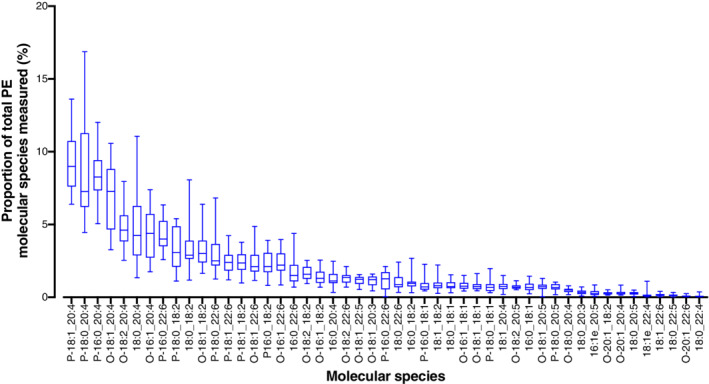
Plasma phosphatidylethanolamine molecular species composition of plasma. Values are proportions in descending order of individual molecular species for n = 20 participants (men plus women, n = 10 per sex). Data points represent individual participants. Bar = mean (range). O,*sn*‐1 alkyl,*sn*‐2 ester species; P,*sn*‐1 alkenyl,*sn*‐2 ester species

Plasma from men and women contained 15 diester PtdEtn molecular species (Table 3), 21 alkyl‐ester PtdEtn molecular species, and 12 alkenyl‐ester PtdEtn molecular species (Table 4). The molecular species profile of plasma PtdEtn for all participants is shown in Fig. 2. The proportion of total diester PtdEtn molecular species did not differ significantly between sexes, while the proportion of total alkyl‐ester species was significantly greater in women than in men. The proportion of total alkenyl‐ester molecular species was significantly greater in men than in women (Table 4).

**Table 3:** Plasma phosphatidylethanolamine *sn*‐1, 2 ester‐linked molecular species composition in men and women

	Proportion of total PtdEtn molecular species (%)		*t*‐Test
Molecular species	Men	Women	*p*
PtdEtn16:0_18:1	0.63 ± 0.09	0.84 ± 0.12	0.162
PtdEtn16:0_18:2	0.99 ± 0.20	0.99 ± 0.07	0.984
PtdEtn16:0_20:4	1.17 ± 0.19	1.43 ± 0.18	0.319
PtdEtn16:0_22:6	1.81 ± 0.34	1.82 ± 0.35	0.988
PtdEtn18:0_18:1	0.75 ± 0.11	0.92 ± 0.10	0.274
PtdEtn18:0_18:2	3.38 ± 0.61	3.33 ± 0.32	0.948
PtdEtn18:0_20:3	0.31 ± 0.06	0.37 ± 0.05	0.446
PtdEtn18:0_20:4	4.29 ± 0.62	5.03 ± 0.84	0.491
PtdEtn18:0_20:5	0.23 ± 0.04	0.30 ± 0.03	0.187
PtdEtn18:0_22:4	0.05 ± 0.01	0.12 ± 0.03	0.076
PtdEtn18:0_22:5	0.13 ± 0.02	0.16 ± 0.02	0.296
PtdEtn18:0_22:6	0.94 ± 0.18	1.17 ± 0.18	0.373
PtdEtn18:1_18:2	0.91 ± 0.17	0.78 ± 0.07	0.483
PtdEtn18:1_20:4	0.77 ± 0.11	0.72 ± 0.08	0.763
PtdEtn18:1_22:6	0.18 ± 0.03	0.16 ± 0.03	0.715
Total	16.54 ± 2.37	18.50 ± 1.86	0.601

Values are mean ± SEM (n = 10 per sex) proportions of individual molecular species in total plasma PtdEtn (*sn*‐1 fatty acid / *sn*‐2 fatty acid). Comparisons of means between sexes were by Student's unpaired *t*‐test with adjustment for multiple testing using the Holm‐Šídák method (all adjusted P values were nonsignificant and are not shown). Molecular species are ranked by increasing number of carbons and degree of unsaturation of the putative *sn*‐1 fatty acid.

**Table 4:** Plasma *sn*‐1 alkyl or *sn*‐1 alkenyl phosphatidylethanolamine molecular species composition in men and women

	Proportion of total PtdEtn molecular species (%)		*t*‐Test	
Molecular species	Men	Women	*p*‐Value	Adjusted *p*‐value
Alkyl molecular species				
PtdEtn O‐16:1_18:1	0.72 ± 0.09	0.92 ± 0.08	0.118	0.745
PtdEtn O‐16:1_18:2	1.27 ± 0.14	1.50 ± 0.17	0.315	0.923
PtdEtn O‐16:1_20:4	3.22 ± 0.43	5.47 ± 0.46	0.002	0.035
PtdEtn O‐16:1_20:5	0.25 ± 0.04	0.35 ± 0.08	0.279	0.566
PtdEtn O‐16:1_22:6	2.01 ± 0.28	2.60 ± 0.23	0.126	0.747
PtdEtn O‐18:0_20:4	0.40 ± 0.05	0.55 ± 0.04	0.035	0.451
PtdEtn O‐18:1_18:1	0.75 ± 0.11	0.81 ± 0.06	0.643	0.994
PtdEtn O‐18:1_18:2	3.29 ± 0.41	3.17 ± 0.31	0.834	0.993
PtdEtn O‐18:1_20:3	0.98 ± 0.14	1.30 ± 0.04	0.041	0.515
PtdEtn O‐18:1_20:4	5.95 ± 0.90	7.68 ± 0.49	0.111	0.739
PtdEtn O‐18:1_20:5	0.58 ± 0.10	0.83 ± 0.07	0.056	0.565
PtdEtn O‐18:1_22:4	0.07 ± 0.01	0.31 ± 0.10	0.041	0.432
PtdEtn O‐18:1_22:5	1.01 ± 0.12	1.28 ± 0.05	0.065	0.566
PtdEtn O‐18:1_22:6	2.31 ± 0.35	2.49 ± 0.26	0.686	0.994
PtdEtn O‐18:2_18:2	1.54 ± 0.15	1.80 ± 0.17	0.275	0.924
PtdEtn O‐18:2_20:4	4.01 ± 0.36	5.48 ± 0.41	0.014	0.025
PtdEtn O‐18:2_20:5	0.67 ± 0.05	0.80 ± 0.05	0.079	0.657
PtdEtn O‐18:2_22:6	1.19 ± 0.08	1.41 ± 0.12	0.161	0.788
PtdEtn O‐20:1_18:2	0.30 ± 0.03	0.30 ± 0.04	0.966	0.994
PtdEtn O‐20:1_20:4	0.23 ± 0.03	0.36 ± 0.06	0.055	0.992
PtdEtn O‐20:1_22:6	0.09 ± 0.02	0.10 ± 0.01	0.745	0.997
Total	30.83 ± 2.99	39.51 ± 1.32	0.021	0.991
				
Alkenyl molecular species				
PtdEtn P‐16:0_18:1	1.06 ± 0.17	0.63 ± 0.08	0.041	>0.999
PtdEtn P‐16:0_18:2	2.73 ± 0.30	1.92 ± 0.21	0.040	>0.999
PtdEtn P‐16:0_20:4	8.28 ± 0.73	8.20 ± 0.51	0.931	>0.999
PtdEtn P‐16:0_22:6	4.61 ± 0.36	3.88 ± 0.25	0.114	>0.999
PtdEtn P‐16:0_22:6	1.17 ± 0.21	1.09 ± 0.24	0.816	>0.999
PtdEtn P‐18:0_18:1	0.95 ± 0.17	0.60 ± 0.08	0.098	>0.999
PtdEtn P‐18:0_18:2	4.05 ± 0.48	2.67 ± 0.32	0.029	>0.999
PtdEtn P‐18:0_20:4	9.70 ± 1.28	7.73 ± 0.81	0.214	>0.999
PtdEtn P‐18:0_20:5	0.77 ± 0.07	0.57 ± 0.07	0.071	>0.999
PtdEtn P‐18:0_22:6	3.41 ± 0.48	2.34 ± 0.30	0.077	>0.999
PtdEtn P‐18:1_18:2	2.81 ± 0.26	2.05 ± 0.18	0.028	>0.999
PtdEtn P‐18:1_20:4	10.11 ± 0.78	8.56 ± 0.39	0.100	>0.999
Total	52.63 ± 4.29	42.34 ± 1.11	0.042	>0.999

Values are mean ± SEM (n = 10 per sex) proportions of individual molecular species in total plasma PtdEtn (*sn*‐1 fatty acid / *sn*‐2 fatty acid). Comparisons of means between sexes were by Student's unpaired *t*‐test with adjustment for multiple testing using the Holm‐Šídák method. Molecular species are ranked by increasing number of carbons and degree of unsaturation of the *sn*‐1 fatty acid.

PtdEtn18:0_20:4, PtdEtn18:0_18:1, and PtdEtn16:0_22:6 together accounted for more than 9.5% of total PtdEtn molecular species in men and women, which did not differ between sexes (Table 3). There were no significant differences between men and women in the proportions of individual diester PtdEtn molecular species (Table 3). The three most abundant alkyl ester PtdEtn molecular species were PtdEtn O‐18:1_20:4, PtdEtn O‐18:2 _20:4, and PtdEtn O‐16: _20:4 which combined accounted for 13.2 ± 1.5% of total PtdEtn molecular species in men and 18.6 ± 1.1% of total PtdEtn molecular species in women, which did not differ significantly between sexes (Table 4). The three most abundant alkenyl‐ester PtdEtn molecular species were PtdEtn P‐18:1_20:4, PtdEtn P‐16:0 _20:4, and PtdEtn P‐18:0_20:4 in both sexes (Table 3). Together these species accounted for 28.1 ± 2.5% of total PtdEtn molecular species in men and 24.5 ± 0.9% of total molecular species in women, which did not differ significantly between sexes (Table 4). The proportion of PtdEtn O‐16:1_20:4 and PtdEtn O‐18:2_20:4 were significantly greater in women (69.9% and 36.7%, respectively) than in men. Sex accounted for 41.8% of the variation in the proportion of PtdEtn O‐16:1 _20:4 (adjusted *r* ^2^ = 0.418; *p* = 0.002) and 25.4% of the variation in the proportion of PtdEtn O‐18:2_20:4 (adjusted *r* ^2^ = 0.0.254; *p* = 0.014). There were no significant differences between sexes in the proportions of other alkenyl ester PtdEtn species (Table 4).

### Effect of Sex on the Proportions of PtdSer and PtdIns Molecular Species in Plasma

Three PtdSer molecular species were identified in plasma from both sexes; PtdSer18:0_18:1, PtdSer18:0_20:4, and PtdSer20:2_20:4 (Table 5). There were no significant differences in the proportions of these molecular species between sexes.

**Table 5:** Plasma phosphatidylserine and phosphatidylinositol molecular species compositions in men and women

		
Molecular species	Men	Women	*t*‐Test
Proportion of total PtdSer molecular species (%)			*p*
PtdSer18:0_18:1	9.53 ± 2.16	21.52 ± 6.56	0.111
PtdSer18:0_20:4	12.74 ± 3.16	13.09 ± 3.35	0.940
PtdSer20:2_20:4	77.73 ± 4.32	65.39 ± 7.85	0.190
			
Proportion of total PtdIns molecular species (%)			
PtdIns16:0_16:1	0.57 ± 0.05	0.60 ± 0.12	0.227
PtdIns16:0_18:1	4.51 ± 0.51	5.48 ± 0.60	0.238
PtdIns16:0_18:2	3.36 ± 0.17	3.57 ± 0.28	0.540
PtdIns16:0_20:3	1.07 ± 0.06	1.08 ± 0.06	0.918
PtdIns16:0_20:4	7.24 ± 0.68	7.08 ± 0.53	0.859
PtdIns16:0_20:4	3.79 ± 0.24	4.33 ± 0.34	0.208
PtdIns18:0_18:1	6.37 ± 0.76	8.26 ± 1.01	0.154
PtdIns18:0_18:2	13.55 ± 1.04	12.67 ± 0.75	0.503
PtdIns18:0_20:3	7.00 ± 0.67	6.77 ± 0.38	0.778
PtdIns18:0_20:4	45.24 ± 1.60	42.91 ± 1.98	0.372
PtdIns18:0_22:5	0.93 ± 0.08	0.88 ± 0.06	0.655
PtdIns18:0_22:6	1.78 ± 0.26	2.09 ± 0.19	0.342
PtdIns18:1_18:2	2.64 ± 0.18	2.27 ± 0.08	0.089
PtdIns18:1_20:4	2.26 ± 0.12	2.00 ± 0.11	0.141

Values are mean ± SEM (n = 10 per sex) proportions of individual molecular species (*sn*‐1 fatty acid / *sn*‐2 fatty acid) in each lipid class. Comparisons of means between sexes were by Student's unpaired *t*‐test with adjustment for multiple testing using the Holm‐Šídák method (all adjusted *p*‐values were nonsignificant and are not shown). Molecular species are ranked by increasing number of carbons and degree of unsaturation of the putative *sn*‐1 fatty acid.

**Fig 3:**
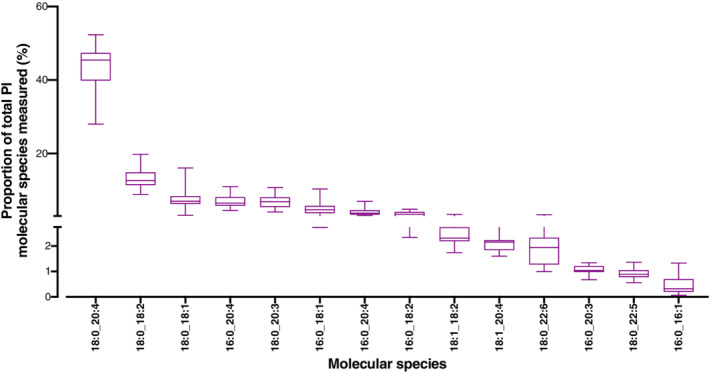
Plasma phosphatidylinositol molecular species composition. Values are proportions in descending order of individual molecular species for n = 20 participants (men plus women, n = 10 per sex). Data points represent individual participants. Bar = mean (range). O,*sn*‐1 alkyl,*sn*‐2 ester species; P,*sn*‐1 alkenyl,*sn*‐2 ester species

Fourteen PtdIns molecular species were identified in men and women (Table 5). The molecular species profile of plasma PtdIns for all participants is shown in Fig. 3. The major PtdIns molecular species in men and women was PtdIns18:0_20:4, which alone accounted for over 42% of total PtdIns molecular species in both sexes. There were no significant differences in the proportions of PtdIns molecular species between sexes (Table 5).

## Discussion

As described previously, the present findings show that the plasma phospholipid pool is composed of a complex mixture of phospholipid classes and molecular species (Burla et al., 2018; Quehenberger et al., 2010), although the total number of molecular species detected in each phospholipid class differed to some extent from those reported here. It is possible that this may reflect the participants in the earlier study, who were of undisclosed sex, geographical differences in habitual diet, and being older (40–50 years old) than those in the current study.

Each phospholipid class was composed of a relatively small number of molecular species that accounted for the majority of each phospholipid class and a larger number of molecular species that individually accounted for less than 1% of total species. As described previously (Inoue et al., 2017), each phospholipid class had a distinct molecular species profile that differed in the diversity of the type and combinations of fatty acids, the presence of *sn*‐1 ether‐linked fatty acids and the number of molecular species (Kawanishi et al., 2018). For example, PtdCho and PtdEtn contained *sn*‐1 alkyl species, while PtdEtn, but not PtdCho, contained *sn*‐1 alkenyl species. Ether‐linked fatty acids were absent from PtdSer and PtdIns. The PtdCho molecular species composition reported here is in agreement with the partial analysis reported previously, for example PtdCho16:0/18:2, PtdCho16:0/18:1, and PtdCho18:0/18:2 were the most abundant PtdCho molecular species in pregnant women (Postle et al., 1995). However, there are marked differences in PtdCho molecular species composition between the current study and Quehenberger et al., 2010. For example, the three most abundant PtdCho species in the present study were PtdCho16:0_18:2, PtdCho16:1_18:1, and PtdCho18:0_18:2, while the three most abundant PtdCho species in Quehenberger et al., 2010 were PtdCho(38:4), PtdCho(36:2), and PtdCho(34:2) which we suggest for comparison were PtdCho18:0_20:4, PtdCho18:1_18:1 or PtdCho18:0_18:2, and PtdCho16:0_18:2 or 16:1_18:1, respectively. The PtdEtn and PtdIns molecular species profiles resembled those reported previously (Gardner et al., 2019; Kawanishi et al., 2018; Quehenberger et al., 2010). However, these studies only reported the total number of carbons and level of unsaturation of each molecular species instead of individual fatty acid combinations thus preventing direct comparison with the present data (Gardner et al., 2019; Kawanishi et al., 2018). Quehenberger et al. (2010) reported 20 PtdSer molecular species compared to three PtdSer molecular species in the present study. However, PtdSer (40:6), which we suggest may have been PtdSer20:2_20:4 or PtdSer18:0_22:6, was the predominant species in both studies. Overall, there is broad agreement between studies about the molecular species compositions of plasma phospholipids and their complexity. However, differences between study designs, including selection of participants and analytical methods, and reporting of molecular species in terms of numbers of total fatty acid carbons and double bonds which suggest alternative combinations of fatty acids for each species, may account, at least in part, for inconsistencies in findings between reports.

The proportions of 20:4n‐6 and 22:6n‐3 are typically 20% higher in total plasma lipids and phospholipids from women compared to men (Lohner et al., 2013). Such sex differences in 20:4n‐6 and 22:6n‐3 concentrations were associated with differences in the concentrations of individual PtdCho and PtdEtn molecular species. In particular, higher 22:6n‐3 concentration in women than men was due entirely to a greater proportion of PtdCho16:0_22:6. The proportion of this species was approximately 64% greater in women than in men. The proportions of other PtdCho species that contained 22:6n‐3 did not differ between sexes. The sum of all PtdCho molecular species that contained 22:6n‐3 showed that the proportion of this fatty acid was significantly greater in women (7.6 ± 0.6 mol%) than in men (6.2 ± 0.5 mol%; adjusted P = 0.005). The magnitude of the difference between sexes for total PtdCho 22:6n‐3 was lower (22.3%) than for PtdCho16:0_22:6 and is similar to that estimated based on analysis of studies that measured total plasma or total phospholipids by Lohner et al. (2013). Thus, it is plausible that PtdCho16:0_22:6 alone may account for the difference in the concentration or proportion of 22:6n‐3 between sexes, but that the magnitude of this difference is reduced by the presence in blood of other 22:6n‐6 ‐containing molecular species that do not exhibit sexual dimorphism.

One previous study investigated the phospholipid composition of young and older men and women (Ishikawa et al., 2014). The findings showed relatively few differences in plasma phospholipid composition between sexes in the young participant group compared to the present study. The primary differences between sexes were higher proportions of individual sphingomyelin molecular species in young women. The proportions of a greater number of phospholipid molecular species differed between sexes in the older participant group (Ishikawa et al., 2014). The only finding that agreed with the present study was that the proportion of PtdCho18:1_22:6 was greater in men than women. The reason for the differences in findings between these studies is not clear, although differences in methodologies and in the ethnicity of the participants may be important.

The concentration of plasma PtdCho16:0/22:6 has been shown to increase significantly during pregnancy in women (Postle et al., 1995) and rodents (Burdge et al., 1993; Childs et al., 2012). In rats, conversion of PtdCho16:0/22:6 to 18:0/22:6 by acyl remodeling decreased with increasing gestational age indicating that this process is regulated by sex hormones. One study has reported that the flux through the PtdEtn N‐methylation pathway was increased in pregnant rats (Chalil et al., 2018), although others have not found this (Burdge et al., 1994). There was no significant difference between sexes in the proportion of PtdEtn16:0_22:6 in the present study. This suggests that unlike rats (Burdge et al., 1994), sex hormones do not appear to alter the composition of the DAG pool destined for incorporation into PtdEtn in humans and that increased N‐methylation of PtdEtn16:0_22:6 is unlikely to account for the greater proportion of PtdCho16:0_22:6 in women than in men. Hepatic acyl remodeling of newly synthesized PtdCho has also been shown to be important in humans (Pynn et al., 2011). Flux through the Lands pathway is reduced in pregnant compared to nonpregnant rats (Burdge et al., 1994). This suggests that one possible explanation for a higher proportion of PtdCho16:0_22:6 in women than in men, is lower hepatic activity of the Lands cycle in women. Moreover, LPAT activity, which confers specificity on acyl‐remodeling (Wang and Tontonoz, 2019), may be a locus of metabolic regulation by sex hormones. If so, greater capacity for PUFA biosynthesis in young women compared to men (Burdge et al., 2002; Burdge and Wootton, 2002) appears to be coordinated with PtdCho biosynthesis by female sex hormones.

A systematic review of 51 studies found that the concentration of 20:4n‐6 was higher in plasma phospholipids from women than men (Lohner et al., 2013). The present findings show that the proportions of PtdEtn O‐16:1_20:4, PtdEtn O‐18:2_20:4, but not of other 20:4n‐6‐containing PtdEtn species, were significantly greater in women than men. This may explain why previous studies that analyzed either plasma total lipids or total phospholipids found a sex difference in 20:4n‐6 concentration (Lohner et al., 2013), but this was not detected when the fatty acid composition of purified plasma PtdCho was analyzed (Bakewell et al., 2006). Ether‐linked PtdEtn molecular species are involved in stabilizing the structures of cell membranes (Dean and Lodhi, 2018), although it is not known if they also stabilize the phospholipid monolayer of lipoproteins. Ether‐linked PtdSer species have been suggested to act as antioxidants (Meikle et al., 2011). If so, association of 20:4n‐6 with ether‐linked PtdEtn species may reduce free radical‐mediated oxidation. The greater overall proportion of *sn*‐1 alkyl‐linked PtdEtn species in women compared to men may confer a higher level of antioxidant protection in the presence of a greater potential for oxidation due to increased amounts of 20:4n‐6 and 22:6n‐3. Synthesis of ether lipids in mammals involves a multi‐step pathway that is incompletely understood (Watschinger and Werner, 2013). To our knowledge, sexual dimorphism in the activity of this pathway has not been reported previously.

PtdIns has been shown to be associated with a VLDL/LDL fraction which suggests that it is specific component of at least some lipoproteins although the molecular species composition was not reported (Sun et al., 2019). It is also possible that plasma PtdIns may reflect contamination of plasma with cell debris. One study reported 14 PtdIns molecular species in human platelets, of which the major species were 38:4 > 32:0 > 38:3 (Mujalli et al., 2018) which can be deduced tentatively to be PtdIns18:0_20:4, PtdIns16:0_16:0, or PtdIns18:0_14:0 and PtdIns18:0_20:3, respectively. Although PtdIns18:0_20:4 is the most abundant species in most cell membranes (Holub, 1986), PtdIns18:0_18:2 and PtdIns16:0_20:4 were the second and third most abundant plasma PtdIns molecular species in the present study. Furthermore, no disaturated PtdIns species were detected in plasma. Thus, although contamination of plasma with membrane PtdIns from platelets or cells cannot be completely excluded, we suggest that any such contamination would be a minor artifact in the analysis and that plasma PtdIns is probably associated primarily with lipoproteins.

PtdSer has been detected in microparticles in blood that have been implicated in hypercoagulation in diabetic kidney disease (Yu et al., 2018), cancer (Lea et al., 2017; Liu et al., 2019; Zhao et al., 2016), and inflammation (Zhao et al., 2016). Whether PtdSer is also an integral component of lipoproteins in humans is not known. PtdSer was not detected in the only previous study which attempted to measured PtdSer in VLDL (Sun et al., 2019) and the present data show that PtdSer accounted for less than 0.02% of plasma total phospholipids. The role of PtdSer as a focus for coagulation may preclude its incorporation into the surface monolayer of lipoproteins.

Although the diets of the participants were not controlled, the rank order of molecular species within each phospholipid class was broadly consistent between individuals thus producing a characteristic molecular species profile for each phospholipid class. Plasma phospholipid molecular species composition is regulated by the specificity of the hepatic synthesis of each phospholipid class and differential incorporation of each class into nascent lipoproteins (Pynn et al., 2011). For example, PtdCho is the major phospholipid class associated with liver‐derived lipoproteins which form the phospholipid monolayer (Nelson & Freeman 1960), while nascent VLDL particles are enriched in PtdEtn which is progressively removed between hepatocyte Golgi apparatus and secretion into blood (Hamilton and Fielding, 1989). The diversity of molecular species between phospholipid classes, while retaining characteristic profiles between individuals, is consistent with the specificity of hepatic phospholipid biosynthesis being a primary determinant of plasma phospholipid molecular species composition (Burdge et al., 1994; Pynn et al., 2011). Demonstration that the proportions of individual plasma molecular species can be influenced by sex, aging (Kawanishi et al., 2018), pregnancy (Postle et al., 1995), and obesity (Donovan et al., 2013) suggests that endocrine factors are an important influence on hepatic phospholipid and lipoprotein metabolism.

The main limitation of this observational study is that it only allowed speculation about the underlying processes instead of providing direct mechanistic insights, for example based on tracer technologies. Such interpretations were based on measurements of total plasma, while it would have been preferable to have analyzed isolated VLDL particles as a more direct representation of hepatic phospholipid metabolism. It was not possible to test directly the effects of differences in phospholipid composition on VLDL structure and function. Additional limitations of this exploratory study include the relatively small sample size, and limited range of ages and ethnicities which constrain the extent to which the findings can be extrapolated to the wider population. Nevertheless, the findings show for the first time that plasma phospholipid composition can be influenced by sex. One future challenge will be to test whether such sexual dimorphism in phospholipid molecular species composition contributes to sex differences in lipoprotein metabolism and health outcomes.

It has been proposed that specific phospholipid molecular species in cell membranes act as “pivot” species which are resistant to hydrolysis and are required for maintaining membrane structure (Dymond et al., 2013). We have suggested that such pivot species may be present in lipoproteins and are required to maintain the structure of the phospholipid monolayer and/or facilitate the activities of lipases (West et al., 2020). Hepatic and/or plasma PtdCho16:0/22:6 concentration in humans and rodents can be modified by sex hormones (Burdge et al., 1994; Chalil et al., 2018; Childs et al., 2012; Postle et al., 1995) and appears to be resistant to postprandial hydrolysis (West et al., 2020). Thus PtdCho16:0/22:6 may be a pivot molecular species in lipoproteins. PtdEtn regulates the curvature of in the inner mitochondrial membranes and is involved in points of membrane contact, by disrupting the PtdCho bilayer due to the inverted hexagonal packing of PtdEtn molecules (Daum and Vance, 1997). PtdIns has also been shown to promote membrane curvature (Mulet et al., 2008), although whether PtdEtn and PtdIns are involved in regulating membrane shape in lipoproteins is not known.

One possible explanation for the large number of plasma phospholipid molecular species is that the overall effect on the biophysical properties of the phospholipid monolayer produces an optimal environment for lipoprotein function including the activities of integral proteins. If so, sex differences in plasma phospholipid classes and molecular species compositions may modify lipoprotein function in a manner that contributes to different patterns of health and disease between men and women.
